# Lactation Duration and the Risk of Subtypes of Stroke Among Parous Postmenopausal Women From the China Kadoorie Biobank

**DOI:** 10.1001/jamanetworkopen.2022.0437

**Published:** 2022-02-25

**Authors:** Ziyang Ren, Qian Yi, Leying Hou, Tzu Tsun Luk, Yiwen Qiu, Wei Xia, Yimin Zhu, Peige Song, Kazem Rahimi

**Affiliations:** 1 School of Public Health and Women’s Hospital, Zhejiang University School of Medicine, Hangzhou, China; 2 School of Nursing, The University of Hong Kong, Hong Kong, China; 3 School of Nursing, Sun Yan-Sen University of Medical Sciences, Guangzhou, China; 4 Department of Epidemiology and Biostatistics, School of Public Health, Zhejiang University School of Medicine, Hangzhou, China; 5 Nuffield Department of Women’s & Reproductive Health, University of Oxford, Oxford, United Kingdom; 6 Deep Medicine Programme, Oxford Martin School, University of Oxford, Oxford, United Kingdom

## Abstract

**Importance:**

Previous studies have reported favorable associations between lactation and cardiovascular diseases. Various stroke subtypes are caused by different pathological processes; however, to date, the associations of lactation duration with different stroke subtypes are less well established.

**Objective:**

To examine the associations of lactation duration with stroke and its subtypes in parous postmenopausal women.

**Design, Setting, and Participants:**

This population-based prospective cohort study included parous postmenopausal women aged 45 to 79 years in the China Kadoorie Biobank (CKB) study at baseline (2004-2008). Lactation duration was counted as lifetime, mean per child, and for the first child. New-onset stroke and its subtypes (ischemic stroke, intracerebral hemorrhage [ICH], and subarachnoid hemorrhage [SAH]) were assessed via disease registries and national health insurance claim databases during follow-up (2008-2015). Data were analyzed from June to December 2021.

**Exposures:**

Lactation duration (lifetime, mean per child, and for the first child).

**Main Outcomes and Measures:**

The main outcomes were total stroke, ischemic stroke, ICH, and SAH. Multivariable Cox regression was applied to calculate the adjusted hazard ratios (aHRs) with 95% CIs for stroke and subtypes.

**Results:**

Of 129 511 parous postmenopausal women (median [IQR] age, 58.3 [54.0-64.6] years) without prior stroke at baseline, 15 721 developed stroke, with median (IQR) lifetime lactation duration of 42.0 (24.0-70.0) months among 13 427 women who had ischemic stroke, 54.0 (36.0-84.0) months among 2567 women who had ICH, and 36.0 (24.0-64.5) months among 284 women with SAH. Compared with parous postmenopausal women who had never lactated, those with lifetime lactation duration of at least 7 months had lower risks of ischemic stroke (aHRs varying from 0.52 [95% CI, 0.50-0.55] to 0.64 [95% CI, 0.59-0.69]) and ICH (aHRs, 0.56 [95% CI, 0.49-0.63] to 0.78 [95% CI, 0.64-0.96]). However, for SAH, such associations were found only in participants with lifetime lactation duration longer than 24 months (aHR, 0.61 [95% CI, 0.47-0.79]). Additionally, women with mean lactation duration per child or lactation duration for the first child of 7 months or longer were less likely to develop stroke (aHRs varying from 0.53 [95% CI, 0.52-0.54] to 0.65 [95% CI, 0.63-0.67]) and its subtypes (aHRs varying from 0.51 [95% CI, 0.30-0.87] to 0.75 [95% CI, 0.69-0.81]).

**Conclusions and Relevance:**

In this cohort study, lactation was significantly associated with a lower risk of stroke, especially ischemic stroke, emphasizing the importance of promoting breastfeeding as a targeted prevention strategy of stroke.

## Key Points

**Question:**

Is lactation duration associated with risk of stroke and its subtypes among parous postmenopausal women?

**Findings:**

In this cohort study of 129 511 parous postmenopausal women aged 45 to 79 years, women who ever lactated, regardless of lactation duration, were at a significantly lower risk of ischemic stroke, intracerebral hemorrhage (except lifetime lactation of <7 months), and subarachnoid hemorrhage (except lactation for lifetime ≤24 months and for per child or the first child <7 months), compared with women who had never lactated.

**Meaning:**

These findings suggest that lactation had a beneficial association for risk of stroke, especially ischemic stroke.

## Teaser

This cohort study examines the association of lactation duration with stroke and its subtypes in parous postmenopausal women from the China Kadoorie Biobank cohort.

## Introduction

Stroke has evolved into an important global health problem and is now the third leading cause of disability adjusted–life years (DALYs) worldwide and the first leading cause of DALYs in China.^1,2^ According to the Global Burden of Disease study 2017, the burden of stroke has grown over the past 3 decades in China, with DALY counts increased by 46.8% and rates of stroke increased by 24.4%.^3,4^ Moreover, from 2015 to 2017, mean costs for a patient with stroke were estimated to be US$1627 for hospitalization and US$691 in out-of-pocket expenses in northeast China,^5^ imposing a heavy financial burden on patients, families, and society. Given the increasing DALYs, subsequent huge societal economic costs, and the devastating consequences of stroke,^6^ primary prevention is of great importance.

Stroke can be divided into various subtypes, including ischemic stroke, intracerebral hemorrhage (ICH), and subarachnoid hemorrhage (SAH). Different stroke subtypes are caused by specific pathogeneses. Ischemic strokes are usually related to reduced blood flow caused by arterial occlusion; ICHs mostly result from cerebral arteries rupture, whereas SAHs are typically due to ruptured aneurysms.^7^ These different mechanisms imply that diverse stroke subtypes may have different sensitivities to risk factors. Previous studies have demonstrated that body mass index (BMI), waist-hip ratio adjusted for BMI, and weight change were significantly associated with ischemic stroke rather than hemorrhagic stroke,^8,9^ which was also suggested by findings from the associations of stroke subtypes with lipids levels and type 2 diabetes.^10,11,12^ Therefore, further attention is needed on exploring specific risk factors for various stroke subtypes.

Lactation is a natural way to provide optimal nutrition to infants.^13^ Exclusive breastfeeding for infants until age 6 months and continued breastfeeding until age 2 years is recommended by the World Health Organization^14^ and the Guideline to Postpartum Health Services in China.^15^ Previous studies have observed a protective association of lactation with maternal health. With regard to this, Stuebe et al^16^ hypothesized that lactation could “reset” the metabolic process that was developed during pregnancy, for example, influencing insulin resistance and blood lipid level, thereby reducing long-term cardiometabolic risks. Emerging studies have also shown that lactation has protective associations against hypertension, diabetes, metabolic syndrome, and cardiovascular diseases (CVDs).^17,18^ Additionally, evidence indicated that women with a lactation duration of 12 months or longer were less likely to develop myocardial infarction, angina, and congestive heart failure^19,20,21^ compared with women who never lactated.

Most previous research has focused on the association between lactation and CVDs. However, few studies have investigated the association between lactation and stroke subtypes. A study conducted in the US suggested that a longer lifetime lactation duration was associated with lower risk of stroke, but the associations of lactation with different pathological subtypes of stroke (eg, ischemic stroke, ICH, and SAH) were not evaluated.^22^ A study by Peters et al^20^ investigated the association between lactation and different stroke subtypes in women, but their participants were a mixture of premenopausal, perimenopausal, and postmenopausal women, which may confound establishing the association between lactation and stroke owing to the fluctuations in endogenous hormones. To fill this knowledge gap, we prospectively evaluated the associations of lactation duration (lifetime lactation duration, mean lactation duration per child, and lactation duration for the first child) with stroke and its subtypes (ischemic stroke, ICH, and SAH) among parous postmenopausal women in the China Kadoorie Biobank (CKB) cohort.

## Methods

This cohort study using the CKB cohort obtained ethics approval from the Ethical Review Committee of the Chinese Center for Disease Control and Prevention and the Oxford Tropical Research Ethics Committee. All participants provided written informed consent. This study is reported following the Strengthening the Reporting of Observational Studies in Epidemiology (STROBE) reporting guideline.

### Study Population

The CKB study is a nationwide, prospective cohort study. Adults aged 30 to 79 years were recruited from 10 regions across China between June 2004 and July 2008, including 5 urban and 5 rural areas. All adults who were permanent residents without a serious disease were invited. The participation rate was more than 98%. Details of the study design and procedures have been reported elsewhere.^23,24,25^

**Figure 1.**
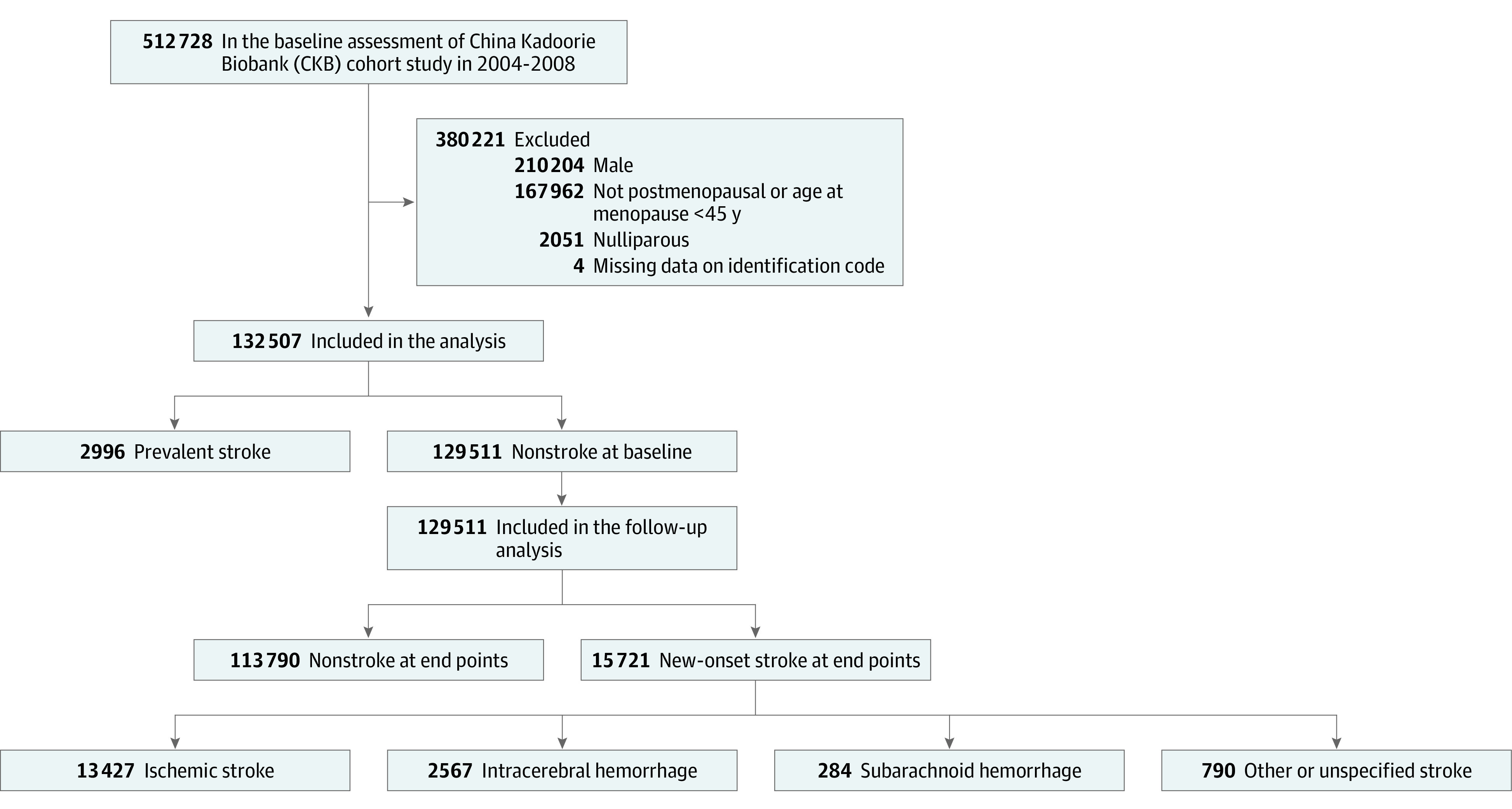
Participant Recruitment Flowchart

Participants completed the baseline survey and were followed-up for nearly 10 years. The study flowchart is shown in Figure 1. Among 512 728 adults recruited at the baseline during 2004 to 2008, men, women who had missing identification codes, women who were not postmenopausal or whose age at menopause was younger than 45 years, who were nulliparous, or who had prevalent stroke at baseline were excluded, leaving 129 511 participants.

### Assessment and Definition of Lactation Duration

At baseline during 2004 to 2008, women completed a detailed questionnaire on their reproductive histories, including live birth counts and lactation duration for each of their children. Lifetime lactation duration was calculated as the sum of the lactation duration for each child; mean lactation duration per child was defined as lifetime lactation duration divided by the number of live births; lactation duration for the first child referred to the lactation duration for women’s first child.^23,24,25^ Lactation duration was categorized as 0, less than 7 months, 7 to 12 months, 13 to 18 months, 19 to 24 months, and longer than 24 months.

### Follow-up for New-Onset Stroke

At baseline, participants self-reported their stroke history through questionnaires while the subtypes were ambiguous. New-onset stroke during follow-up was assessed by hospitals and further linked to the disease registries and new national health insurance claim databases in China.^23,24,25^ All strokes were coded with the *International Statistical Classification of Diseases and Related Health Problems, Tenth Revision* (*ICD-10*) (I63 for ischemic stroke; I61 for ICH; and I60 for SAH).^26,27^ Annual home visits, medical records, and brain imaging reports were further used to confirm the accuracy of diagnoses by clinical specialists.

### Measurement of Covariates

Information on age, age at menopause, gravidity, live birth counts, habitual residence, highest education completed, household income, marital status, occupation, physical activity, smoking and drinking history, passive smoking exposure, medical history (diabetes, hypertension, cancer), and medication history (contraceptive pills, anticoagulation therapy, hypolipidemic therapy) was collected through questionnaires at baseline during 2004 to 2008 by uniformly trained health workers.^23,24,25^ Meanwhile, physical measurements and blood biomarkers were also recorded, including standing height, weight, waist circumference, blood pressure, and blood glucose.^24^ Physical activity was measured by metabolic equivalent task hours per day spent in working and a set of leisure activities. Diabetes was defined as fasting glucose 126.13 mg/dL or greater, random glucose 198.20 mg/dL or greater (to convert to millimoles per liter, multiply by 0.0555), or self-reported diagnosis or receiving treatment; hypertension was defined as blood pressure 140/90 mm Hg or greater or self-reported diagnosis or receiving treatment.

### Statistical Analysis

The characteristics of included participants by stroke status at baseline were described in the form of means and SDs for normally distributed continuous variables, medians and IQRs for nonnormally distributed continuous variables, and with number and percentage for categorical variables.

First, the incidence density of new-onset total stroke and its 3 subtypes was calculated as the number of events per 100 000 person-years. Furthermore, Cox proportional hazards models with age as the time scale and the strata function were used to investigate the association of lactation duration (lifetime lactation duration, mean lactation duration per child, and lactation duration for the first child) with new-onset stroke and its subtypes during follow-up. The age of ischemic stroke, ICH, SAH, or other unspecified strokes, whichever happened first, was used to determine the age of new-onset stroke. Hazard ratios (HRs) and 95% CIs were estimated using floating absolute risk. The floating approaches allow acceptable comparisons between any 2 exposure groups, which decreases undesired correlation between coefficients.^28,29,30^ Model 1 was age-adjusted. Model 2 was further adjusted for gravidity (≤3, 4, ≥5), age at menopause, live birth counts (1, 2, ≥3), diabetes status (yes/no), hypertension status (yes/no), history of cancer (yes/no), using contraceptive pills (yes/no), anticoagulation therapy (yes/no), hypolipidemic therapy (yes/no), highest education completed (≤primary school, middle or high school, ≥technical school/college), household income (≤4999 [≤US$789.98], 5000-19 999 [US$190.14-$3160.40], or ≥20 000 [US$3160.56] yuan), marital status (yes/no), occupation (farmer or worker, sales and others, professionals, retired, and others), and residence (urban/rural) based on Model 1. Model 3 was further adjusted for metabolic equivalent task hours per day, smoking status (never/ever), exposure to passive smoking (yes/no), drinking history (never/ever) based on Model 2. Model 4 was further adjusted for BMI and waist circumference based on Model 3. Restricted cubic splines (RCS) were constructed to determine the dose-risk association of lactation duration with stroke and its subtypes. Age-stratified Cox and age-stratified RCS were performed to manage the wide age range.

Second, sensitivity analyses were conducted for longitudinal analyses by excluding participants who were using cardiovascular drugs (eg, angiotensin-converting enzyme inhibitors, β-blocker, diuretics, aspirin, calcium channel blocker, and statins) at baseline, who had CVDs (eg, coronary heart disease, kidney disease, and rheumatic heart disease) at baseline, or who developed more than 1 subtype of stroke during follow-up separately to explore how these factors might confound the models’ performance. Finally, cross-sectional sensitivity analysis using the CKB data set from baseline (2004-2008) was conducted with a logistic regression model to approximately reflect the cumulative association (odds ratio with 95% CI) between lactation duration and stroke occurred before the baseline. The stepwise adjustments were made, as those used in the Cox proportional hazards regression.

All analyses were conducted using SAS statistical software version 9.4 (SAS Institute). All analyses were 2-sided, and a 95% CI that did not include 1.00 was considered statistically significant. Data were analyzed from June to December 2021.

## Results

A total of 129 511 parous postmenopausal women (median [IQR] age, 58.3 [54.0-64.6] years) without prior stroke at baseline were included, of whom 15 721 developed strokes (13 427 women with ischemic stroke, 2567 women with ICH, and 284 women with SAH) during 10 years of follow-up. The baseline characteristics of parous postmenopausal women are shown in eTable 1 in the Supplement. The median (IQR) lifetime lactation duration was 42.0 (24.0-70.0) months for women with ischemic stroke, 54.0 (36.0-84.0) months for women with ICH, and 36.0 (24.0-64.5) months for women with SAH. In terms of the median (IQR) lactation duration per child, women with ischemic stroke lactated for 12.7 (12.0-19.5) months, women with ICH lactated for 15.0 (12.0-22.0) months, and women with SAH lactated for 12.0 (12.0-18.0) months. Moreover, for their first child, participants who developed ischemic stroke breastfed for a median (IQR) of 12.0 (12.0-18.0) months, women who developed ICH breastfed for a median (IQR) of 13.0 (12.0-24.0) months, and women with SAH breastfed for 12.0 (12.0-18.0) months.

Among included women, the incidence of total stroke was 1411.8 per 100 000 person-years, 1200.9 per 100 000 person-years for ischemic stroke, 221.3 per 100 000 person-years for ICH, and 24.4 per 100 000 person-years for SAH. Furthermore, eTable 2 and eFigure 1 in the Supplement show the incidence of stroke and its subtypes by lactation duration.

**Figure 2.**
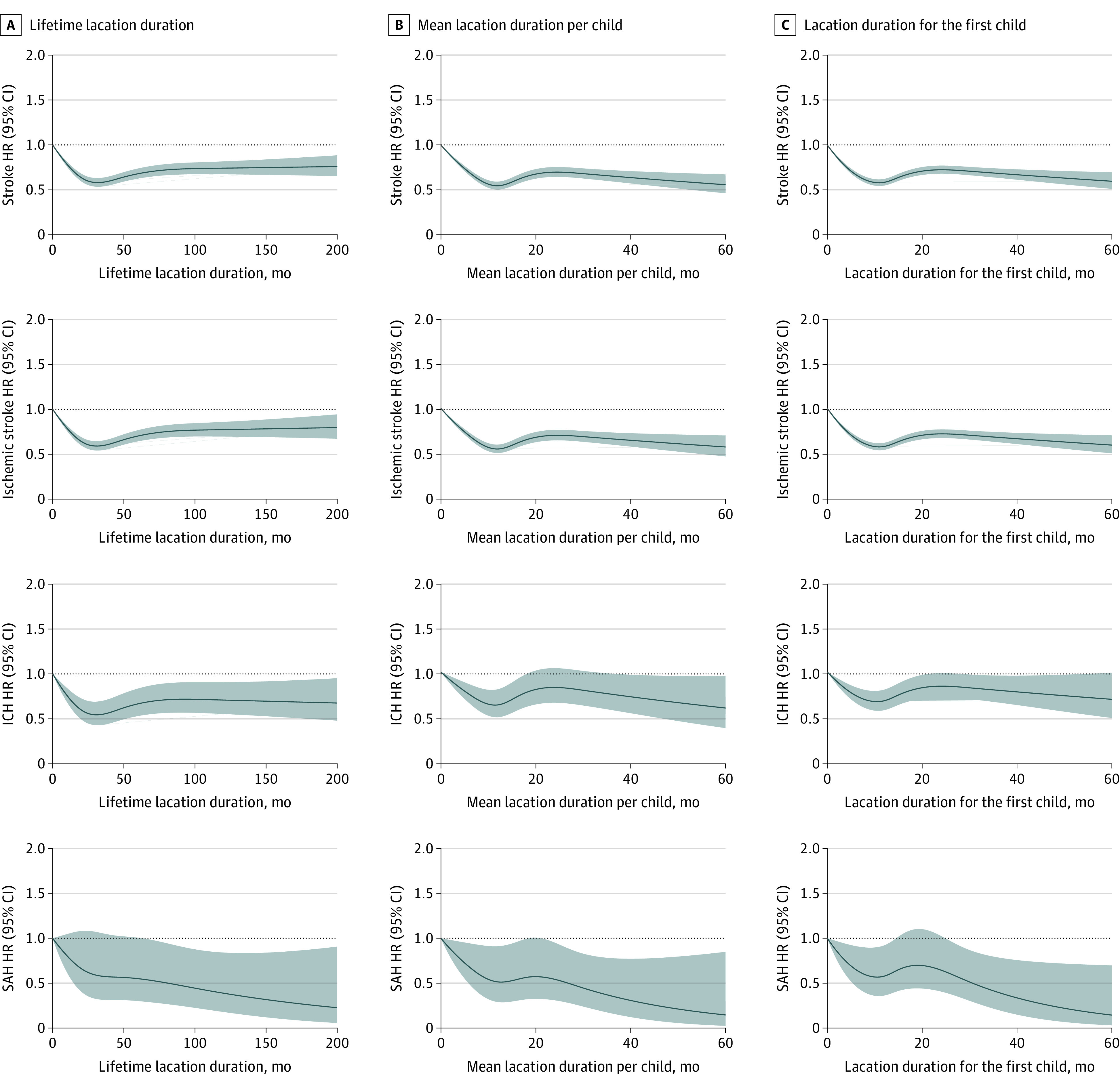
The Dose-Risk Association of Lactation Duration With Stroke and Its Subtypes in Parous Postmenopausal Women Hazard ratios (HRs) were adjusted for age, gravidity, age of menopause, live birth counts, diabetes, hypertension, cancer, taking contraceptive pills, anticoagulation therapy, hypolipidemic therapy, education, income, marital status, occupation, residence, smoking, passive smoking, drinking, metabolic equivalent, body mass index, and waist circumference. ICH indicates intracerebral hemorrhage; SAH, subarachnoid hemorrhage.

As shown in Table 1, there was a reduced risk of ischemic stroke among postmenopausal women who had breastfed for 7 to 12 months (fully adjusted HR, 0.61 [95% CI, 0.57-0.66]), 13 to 18 months (fully adjusted HR, 0.64 [95% CI, 0.59-0.69]), 19 to 24 months (fully adjusted HR 0.52 [95% CI, 0.50-0.55]), and longer than 24 months (fully adjusted HR, 0.56 [95% CI, 0.54-0.58]) in a lifetime. Similarly lifetime breastfeeding of at least 7 months was associated with reduced risk of ICH (7-12 months: fully adjusted HR, 0.78 [95% CI, 0.64-0.96]; 13-18 months: fully adjusted HR, 0.64 [95% CI, 0.51-0.81]; 19-24 months: fully adjusted HR, 0.56 [95% CI, 0.49-0.63]; >24 months: fully adjusted HR, 0.60 [95% CI, 0.55-0.65]) compared with parous postmenopausal women who had never breastfed. Similar results were observed in women who had breastfed for any duration per child with ischemic stroke (fully adjusted HRs varying from 0.53 [95% CI, 0.51-0.54] to 0.75 [95% CI, 0.69-0.81]) and ICH (fully adjusted HRs varying from 0.55 [95% CI, 0.51-0.59] to 0.73 [95% CI, 0.68-0.79]) compared with those who had never breastfed (Table 2). Corresponding results were also found for risk of ischemic stroke in women who had breastfed for their first child (fully adjusted HRs varying from 0.53 [95% CI, 0.52-0.55] to 0.65 [95% CI, 0.61-0.69]) and for ICH (fully adjusted HRs varying from 0.65 [95% CI, 0.61-0.69] to 0.81 [95% CI, 0.74-0.88]) (Table 3). Despite the fact that the associations of mean lactation duration per child and lactation duration for the first child with SAH and were similar to those in ischemic stroke and ICH, only participants with lifetime lactation duration of longer than 24 months were found to have a lower risk of SAH (fully adjusted HR, 0.61 [95% CI, 0.47-0.79]). The association of lactation duration with ischemic stroke and ICH remained unchanged when stratified by age (eTables 3-5 in the Supplement). Furthermore, the RCS curve (Figure 2) and age-stratified RCS curve (eFigures 2-4 in the Supplement) indicated that lifetime lactation was associated with a reduced risk of ischemic stroke, ICH, and SAH, as were mean lactation duration and lactation duration for the first child.

**Table 1.** Association of Lifetime Lactation Duration With Risk of Stroke and Subtypes Among Parous Postmenopausal Women

Outcome	**HR (95% CI)**					
**0 mo (n =** 2553)	<**7 mo (n =** 2652)	**7**-**12 mo (n =** 12 648)	**13**-**18 mo (n =** 7648)	**19**-**24 mo (n =** 20 694)	>**24 mo (n =** 83 316)
**Total stroke**						
Events, No.	431	294	989	746	1862	11 399
Model 1^a^	1.00 (0.91-1.10)	0.85 (0.76-0.95)	0.62 (0.58-0.66)	0.62 (0.58-0.67)	0.41 (0.40-0.43)	0.37 (0.36-0.38)
Model 2^b^	1.00 (0.91-1.10)	0.81 (0.72-0.91)	0.62 (0.58-0.66)	0.65 (0.61-0.70)	0.52 (0.50-0.54)	0.56 (0.54-0.58)
Model 3^c^	1.00 (0.91-1.10)	0.81 (0.72-0.91)	0.62 (0.58-0.67)	0.66 (0.62-0.71)	0.53 (0.50-0.55)	0.56 (0.55-0.58)
Model 4^d^	1.00 (0.91-1.10)	0.81 (0.73-0.91)	0.63 (0.58-0.67)	0.66 (0.61-0.71)	0.53 (0.50-0.55)	0.56 (0.54-0.58)
**Ischemic stroke**						
Events, No.	395	267	899	665	1657	9544
Model 1^a^	1.00 (0.91-1.10)	0.85 (0.75-0.96)	0.62 (0.58-0.67)	0.61 (0.56-0.66)	0.40 (0.39-0.42)	0.34 (0.33-0.35)
Model 2^b^	1.00 (0.91-1.10)	0.79 (0.70-0.89)	0.60 (0.56-0.65)	0.63 (0.59-0.68)	0.51 (0.49-0.54)	0.56 (0.54-0.59)
Model 3^c^	1.00 (0.91-1.10)	0.79 (0.70-0.89)	0.61 (0.57-0.65)	0.64 (0.60-0.70)	0.52 (0.50-0.55)	0.57 (0.55-0.59)
Model 4^d^	1.00 (0.91-1.10)	0.80 (0.71-0.90)	0.61 (0.57-0.66)	0.64 (0.59-0.69)	0.52 (0.50-0.55)	0.56 (0.54-0.58)
**Intracerebral hemorrhage**						
Events, No.	43	25	103	69	219	2108
Model 1^a^	1.00 (0.74-1.35)	0.73 (0.49-1.08)	0.64 (0.53-0.78)	0.57 (0.45-0.72)	0.50 (0.44-0.57)	0.71 (0.67-0.75)
Model 2^b^	1.00 (0.74-1.35)	0.85 (0.57-1.25)	0.78 (0.63-0.95)	0.63 (0.50-0.80)	0.55 (0.48-0.62)	0.60 (0.55-0.64)
Model 3^c^	1.00 (0.74-1.35)	0.85 (0.57-1.25)	0.78 (0.64-0.96)	0.64 (0.50-0.81)	0.56 (0.49-0.63)	0.60 (0.55-0.65)
Model 4^d^	1.00 (0.74-1.35)	0.83 (0.56-1.24)	0.78 (0.64-0.96)	0.64 (0.51-0.81)	0.56 (0.49-0.63)	0.60 (0.55-0.65)
**Subarachnoid hemorrhage**						
Events, No.	6	8	17	18	48	187
Model 1^a^	1.00 (0.45-2.23)	1.52 (0.76-3.04)	0.68 (0.42-1.12)	0.98 (0.62-1.57)	0.73 (0.55-0.97)	0.48 (0.40-0.57)
Model 2^b^	1.00 (0.45-2.23)	1.45 (0.72-2.92)	0.66 (0.40-1.09)	1.01 (0.63-1.61)	0.82 (0.62-1.07)	0.60 (0.47-0.78)
Model 3^c^	1.00 (0.45-2.23)	1.44 (0.71-2.91)	0.67 (0.40-1.10)	1.02 (0.64-1.63)	0.83 (0.63-1.09)	0.61 (0.47-0.78)
Model 4^d^	1.00 (0.45-2.23)	1.43 (0.71-2.89)	0.66 (0.40-1.10)	1.02 (0.64-1.63)	0.83 (0.63-1.09)	0.61 (0.47-0.79)

Abbreviation: HR, hazard ratio.

^a^ Adjusted for age.

^b^ Further adjusted for gravidity, age of menopause, live birth counts, diabetes, hypertension, cancer, using contraceptive pills, anticoagulation therapy, hypolipidemic therapy, education, income, marital status, occupation, and residence based on model 1.

^c^ Further adjusted for smoking, passive smoking, drinking, and metabolic equivalent based on model 2.

^d^ Further adjusted for body mass index and waist circumference based on model 3.

**Table 2.** Association of Mean Lactation Duration per Child With the Risk of Stroke and Subtypes Among Parous Postmenopausal Women

Outcome	**HR (95% CI)**					
**0 mo (n =** 2553**)**	<**7 mo (n =** 5674)	**7**-**12 mo (n =** 57 602**)**	**13**-**18 mo (n =** 30 431**)**	**19**-**24 mo (n =** 21 557**)**	>**24 mo (n =** 11 694**)**
**Total stroke**						
Events, No.	431	754	6280	3813	2932	1511
Model 1^a^	1.00 (0.91-1.10)	0.64 (0.60-0.69)	0.39 (0.38-0.40)	0.41 (0.40-0.42)	0.41 (0.40-0.43)	0.41 (0.39-0.43)
Model 2^b^	1.00 (0.91-1.10)	0.75 (0.70-0.80)	0.52 (0.51-0.54)	0.60 (0.58-0.62)	0.64 (0.62-0.67)	0.64 (0.61-0.68)
Model 3^c^	1.00 (0.91-1.10)	0.75 (0.70-0.81)	0.53 (0.52-0.54)	0.61 (0.59-0.62)	0.64 (0.62-0.67)	0.64 (0.61-0.67)
Model 4^d^	1.00 (0.91-1.10)	0.75 (0.70-0.81)	0.53 (0.52-0.54)	0.60 (0.58-0.62)	0.64 (0.61-0.66)	0.63 (0.60-0.66)
**Ischemic stroke**						
Events, No.	395	669	5489	3232	2386	1256
Model 1^a^	1.00 (0.91-1.10)	0.62 (0.58-0.67)	0.38 (0.37-0.39)	0.38 (0.37-0.39)	0.37 (0.35-0.38)	0.38 (0.36-0.40)
Model 2^b^	1.00 (0.91-1.11)	0.74 (0.69-0.80)	0.52 (0.51-0.53)	0.60 (0.58-0.62)	0.63 (0.61-0.66)	0.66 (0.62-0.69)
Model 3^c^	1.00 (0.91-1.11)	0.75 (0.69-0.81)	0.53 (0.51-0.54)	0.61 (0.59-0.63)	0.63 (0.61-0.66)	0.65 (0.62-0.69)
Model 4^d^	1.00 (0.91-1.11)	0.75 (0.69-0.81)	0.53 (0.51-0.54)	0.60 (0.58-0.62)	0.62 (0.60-0.65)	0.64 (0.60-0.68)
**Intracerebral hemorrhage**						
Events, No.	43	84	857	666	624	293
Model 1^a^	1.00 (0.74-1.35)	0.72 (0.58-0.90)	0.54 (0.51-0.58)	0.71 (0.66-0.76)	0.86 (0.80-0.93)	0.78 (0.70-0.88)
Model 2^b^	1.00 (0.74-1.35)	0.70 (0.56-0.87)	0.55 (0.51-0.59)	0.63 (0.58-0.68)	0.72 (0.67-0.78)	0.62 (0.55-0.70)
Model 3^c^	1.00 (0.74-1.35)	0.70 (0.57-0.87)	0.55 (0.51-0.59)	0.63 (0.58-0.68)	0.73 (0.67-0.79)	0.62 (0.55-0.70)
Model 4^d^	1.00 (0.74-1.35)	0.70 (0.56-0.87)	0.55 (0.51-0.59)	0.63 (0.59-0.68)	0.73 (0.68-0.79)	0.63 (0.56-0.71)
**Subarachnoid hemorrhage**						
Events, No.	6	16	127	67	44	24
Model 1^a^	1.00 (0.45-2.23)	0.99 (0.61-1.62)	0.60 (0.50-0.71)	0.55 (0.43-0.70)	0.48 (0.35-0.64)	0.50 (0.33-0.74)
Model 2^b^	1.00 (0.44-2.25)	1.11 (0.67-1.83)	0.72 (0.60-0.87)	0.69 (0.55-0.88)	0.63 (0.47-0.85)	0.63 (0.42-0.95)
Model 3^c^	1.00 (0.44-2.25)	1.11 (0.68-1.83)	0.73 (0.60-0.88)	0.70 (0.55-0.88)	0.64 (0.47-0.87)	0.63 (0.42-0.95)
Model 4^d^	1.00 (0.44-2.25)	1.11 (0.67-1.83)	0.73 (0.60-0.88)	0.70 (0.55-0.89)	0.65 (0.48-0.87)	0.64 (0.42-0.96)

Abbreviation: HR, hazard ratio.

^a^ Adjusted for age.

^b^ Further adjusted for gravidity, age of menopause, live birth counts, diabetes, hypertension, cancer, using contraceptive pills, anticoagulation therapy, hypolipidemic therapy, education, income, marital status, occupation, and residence based on model 1.

^c^ Further adjusted for smoking, passive smoking, drinking, and metabolic equivalent based on model 2.

^d^ Further adjusted for body mass index and waist circumference based on model 3.

**Table 3.** Association of Lactation Duration for the First Child With the Risk of Stroke and Subtypes Among Parous Postmenopausal Women

Outcome	**HR (95% CI)**					
**0 mo (n =** 5626**)**	<**7 mo (n =** 8188 **)**	**7**-**12 mo (n =** 62 879**)**	**13**-**18 mo (n =** 25 422**)**	**19**-**24 mo (n =** 19 394**)**	>**24 mo (n =** 8002 **)**
**Total stroke**						
Events, No.	883	1099	6923	3240	2591	985
Model 1^a^	1.00 (0.94-1.07)	0.72 (0.68-0.76)	0.54 (0.53-0.55)	0.61 (0.59-0.63)	0.55 (0.53-0.58)	0.54 (0.51-0.57)
Model 2^b^	1.00 (0.94-1.07)	0.66 (0.62-0.70)	0.54 (0.53-0.55)	0.65 (0.63-0.68)	0.65 (0.62-0.67)	0.62 (0.58-0.66)
Model 3^c^	1.00 (0.94-1.07)	0.66 (0.62-0.70)	0.54 (0.53-0.56)	0.66 (0.63-0.68)	0.65 (0.62-0.67)	0.61 (0.58-0.65)
Model 4^d^	1.00 (0.94-1.07)	0.66 (0.62-0.70)	0.54 (0.53-0.56)	0.65 (0.63-0.67)	0.64 (0.62-0.67)	0.61 (0.57-0.65)
**Ischemic stroke**						
Events, No.	774	961	6002	2757	2118	815
Model 1^a^	1.00 (0.93-1.07)	0.72 (0.68-0.77)	0.54 (0.52-0.55)	0.59 (0.57-0.62)	0.52 (0.50-0.54)	0.51 (0.48-0.55)
Model 2^b^	1.00 (0.93-1.07)	0.65 (0.61-0.69)	0.53 (0.51-0.54)	0.65 (0.63-0.67)	0.63 (0.60-0.66)	0.62 (0.57-0.66)
Model 3^c^	1.00 (0.93-1.07)	0.65 (0.61-0.69)	0.53 (0.52-0.55)	0.65 (0.63-0.67)	0.63 (0.60-0.66)	0.61 (0.57-0.65)
Model 4^d^	1.00 (0.93-1.07)	0.65 (0.61-0.69)	0.53 (0.52-0.55)	0.64 (0.62-0.67)	0.62 (0.6-0.65)	0.60 (0.56-0.64)
**Intracerebral hemorrhage**						
Events, No.	120	150	1011	549	548	189
Model 1^a^	1.00 (0.84-1.20)	0.72 (0.61-0.84)	0.58 (0.55-0.62)	0.75 (0.69-0.81)	0.84 (0.77-0.91)	0.74 (0.64-0.86)
Model 2^b^	1.00 (0.83-1.20)	0.75 (0.64-0.88)	0.65 (0.61-0.70)	0.75 (0.69-0.81)	0.81 (0.74-0.88)	0.70 (0.61-0.81)
Model 3^c^	1.00 (0.83-1.20)	0.74 (0.63-0.87)	0.65 (0.61-0.70)	0.74 (0.68-0.81)	0.81 (0.74-0.88)	0.70 (0.60-0.81)
Model 4^d^	1.00 (0.83-1.20)	0.74 (0.63-0.87)	0.65 (0.61-0.69)	0.75 (0.69-0.81)	0.81 (0.74-0.88)	0.70 (0.60-0.81)
**Subarachnoid hemorrhage**						
Events, No.	14	20	133	60	43	14
Model 1^a^	1.00 (0.59-1.69)	0.82 (0.53-1.27)	0.65 (0.55-0.77)	0.70 (0.55-0.90)	0.60 (0.44-0.81)	0.49 (0.29-0.84)
Model 2^b^	1.00 (0.59-1.69)	0.78 (0.50-1.21)	0.64 (0.54-0.76)	0.70 (0.55-0.91)	0.64 (0.47-0.87)	0.51 (0.30-0.87)
Model 3^c^	1.00 (0.59-1.69)	0.79 (0.51-1.22)	0.65 (0.54-0.77)	0.71 (0.55-0.91)	0.65 (0.48-0.89)	0.51 (0.30-0.87)
Model 4^d^	1.00 (0.59-1.69)	0.79 (0.51-1.22)	0.64 (0.54-0.77)	0.71 (0.55-0.92)	0.65 (0.48-0.89)	0.51 (0.30-0.87)

Abbreviation: HR, hazard ratio.

^a^ Adjusted for age.

^b^ Further adjusted for gravidity, age of menopause, live birth counts, diabetes, hypertension, cancer, using contraceptive pills, anticoagulation therapy, hypolipidemic therapy, education, income, marital status, occupation, and residence based on model 1.

^c^ Further adjusted for smoking, passive smoking, drinking, and metabolic equivalent based on model 2.

^d^ Further adjusted for body mass index and waist circumference based on model 3.

The results were similar in the longitudinal sensitivity analyses, in which participants who were using cardiovascular drugs at baseline or had CVDs or participants who developed more than 1 subtype of stroke during follow-up were excluded (eTable 6-8 in the Supplement). The findings from cross-sectional sensitivity analysis also reflected similar significant cumulative associations of lactation duration and prevalent strokes (eTable 9 and eTable 10 in the Supplement).

## Discussion

To our knowledge, this cohort study is the first to explore whether the associations of lactation duration with stroke vary in different etiological subtypes of stroke. We found an inverse association of lactation with stroke risk. Specifically, parous postmenopausal women with lifetime lactation duration of at least 7 months had lower risks of ischemic stroke and ICH compared with women who never lactated. However, for SAH, such associations were found only in participants with lifetime lactation duration of longer than 24 months. In addition, we found that those with mean lactation duration per child or lactation duration for the first child of at least 7 months were less likely to develop stroke and its subtypes.

Our findings extend results from previous studies of longer lifetime lactation duration in association with lower risks of new-onset stroke.^20,22^ Previous studies hypothesized that a longer lactation duration could increase women’s energy consumption, improve glucose and lipid metabolism, and therefore reduce the risk of CVDs.^31,32,33^ For example, participants who lactated for more than 10 months had improved insulin sensitivity and were less likely to develop impaired glucose intolerance compared with those who lactated for less than 10 months.^31^

Specifically, we found that lactation was associated with a reduced risk of ischemic stroke. Ischemic stroke is caused by a transient or permanent reduction of blood flow, typically by embolic or thrombotic occlusion,^7^ and may be influenced by a series of endocrine and metabolic factors.^34,35^ Breastfeeding has shown protective associations against the development of atherosclerotic disease, namely thrombotic or embolic events, which are usually the cause of ischemic stroke.^36^ Furthermore, lactation can stimulate the secretion of oxytocin, which affects behavioral and neuroendocrine stress responses through stress and the corticotrophin-releasing factor system of the brain,^37,38^ thereby reducing inflammation, repairing myocardial damage, and treating systemic diseases, such as atherosclerosis, diabetes, and hypertension.^39,40,41^ Other hormones, like estrogens and progesterone, have also been reported to be associated with lactation. Previous studies found that estrogen and progesterone therapy was associated with elevated ischemic stroke risk.^42,43,44^ Estrogen and progesterone levels remain low in lactating women until weaning,^45^ contributing to the protective association of lactation with stroke. In addition, given that maternal adrenocorticotropic hormone and cortisol levels decline rapidly when breastfeeding,^45,46^ lactation can downregulate high cortisol levels and hypothalamic-pituitary-adrenal axis activity after delivery,^47^ which may further introduce favorable cardiovascular outcomes and reduced risks of CVDs, like ischemic stroke.^48,49^ Given that ICH is generally caused by deep perforating vasculopathy due to high blood pressure,^50^ which is also a cardiovascular-related issue, similar findings from the association between lactation duration and ICH could be interpreted. However, the association between lifetime lactation and SAH was not as strong as that with ischemic stroke and ICH. SAH is typically caused by a ruptured aneurysm and head trauma, so the role of cardiometabolic risk, which lactation could reduce, may be less prominent in SAH.^51,52^

To our knowledge, this is the first large-scale population-based cohort study to investigate the association of lifetime lactation duration, mean lactation duration per child, and lactation duration for the first child with specific stroke subtypes, including ischemic stroke, ICH, and SAH, in parous postmenopausal women in China. We found that using total stroke as the outcome in epidemiological research may sometimes neutralize the specific associations of exposures (lactation in this study) with various stroke subtypes, underlining the necessity of differentiating stroke by pathogenesis. The large sample size from 10 diverse Chinese regions and high follow-up rate strengthen the reliability and generalizability of our findings. In addition, the diagnoses of stroke subtypes were carried out by professional health workers, so the accuracy of stroke diagnosis and classification could be considered highly reliable. Moreover, since the hazard of the stroke changes more as a function of age than as a function of time-on-study, we used age as the time scale in our Cox models, which has been proven to be accurate when analyzing cohort data. Additionally, we controlled the potential confounding of a wide age range by stratifying age groups, and a set of sensitivity analyses were conducted in our study to show the robustness of our results.

### Limitations

Our study has several limitations. We excluded women who had developed stroke before the arbitrary baseline age in longitudinal analyses, which may cause differential bias in those who were older at baseline. Furthermore, information on risk factors regarding lactation duration was mostly collected based on self-reported questionnaires, which could also result in recall bias. Given we have no relevant data, pregnancy-related morbidity, perinatal outcomes, bleeding disorders, atrial fibrillation, and blood lipid factors were not considered as covariates.

## Conclusions

In this cohort study, we found that parous postmenopausal women who had previously lactated had a lower risk of developing stroke, especially ischemic stroke, than those who had never lactated. Our findings also suggest that it is crucial to distinguish different stroke subtypes and emphasize the importance of promoting breastfeeding as a targeted prevention strategy of specific strokes. According to the National Survey of Factors Influencing Breastfeeding conducted in China in 2019,^53^ the exclusive breastfeeding rate for infants within 6 months in China is 29.2%, much lower than the global mean of 43% in low-income countries and 37% in middle-income countries. Our findings underscore the importance of lactation and urge policy makers and the general public to pay more attention to breastfeeding.

## Notes

Supplement. **eTable 1.** Characteristics of Parous Postmenopausal Women Without Prior Stroke at Baseline **eTable 2.** Incidence Density of Stroke And Its Subtypes Among Parous Postmenopausal Women **eTable 3.** Lifetime Lactation Duration and the Risk of Stroke and Subtypes Among Parous Postmenopausal Women: Age-Stratified Multivariate Cox Regression **eTable 4.** Mean Lactation Duration per Child and the Risk of Stroke and Subtypes Among Parous Postmenopausal Women: Age-Stratified Multivariate Cox Regression **eTable 5.** Lactation Duration for the First Child and the Risk of Stroke and Subtypes Among Parous Postmenopausal Women: Age-Stratified Multivariate Cox Regression **eTable 6.** Sensitivity Analysis: Multivariate Cox Regression Excluding Participants Who Were Taking Cardiovascular Drugs at Baseline **eTable 7.** Sensitivity Analysis: Multivariate Cox Regression Excluding Participants Who Had Cardiovascular Diseases at Baseline **eTable 8.** Sensitivity Analysis: Multivariate Cox Regression Excluding Participants Who Developed More Than 1 Subtype of Stroke During Follow-up **eTable 9.** Characteristics of Parous Postmenopausal Women at Baseline **eTable 10.** Sensitivity Analysis: Lactation Duration and Prevalent Stroke Among Parous Postmenopausal Women in the CKB Study: Logistic Regression at Baseline **eFigure 1.** Incidence Density of Stroke and its Subtypes **eFigure 2.** The Age-Stratified Dose-Risk Association of Lifetime Lactation Duration With Stroke and Its Subtypes in Parous Postmenopausal Women **eFigure 3.** The Age-Stratified Dose-Risk Association of Mean Lactation Duration Per Child With Stroke and Its Subtypes in Parous Postmenopausal Women **eFigure 4.** The Age-Stratified Dose-Risk Association of Lactation Duration for the First Child With Stroke and Its Subtypes in Parous Postmenopausal Women
