# Supplementary material for: Lactation Duration and the Risk of Subtypes of Stroke Among Parous Postmenopausal Women From the China Kadoorie Biobank
Source: JAMA Netw Open. 2022 Feb 25;5(2):e220437. doi: 10.1001/jamanetworkopen.2022.0437 (PMC8881773; doi:10.1001/jamanetworkopen.2022.0437)
Supplement: Supplement. — eTable 1. Characteristics of Parous Postmenopausal Women Without Prior Stroke at Baseline eTable 2. Incidence Density of Stroke And Its Subtypes Among Parous Postmenopausal Women eTable 3. Lifetime Lactation Duration and the Risk of Stroke and Subtypes Among Parous Postmenopausal Women: Age-Stratified Multivariate Cox Regression eTable 4. Mean Lactation Duration per Child and the Risk of Stroke and Subtypes Among Parous Postmenopausal Women: Age-Stratified Multivariate Cox Regression eTable 5. Lactation Duration for the First Child and the Risk of Stroke and Subtypes Among Parous Postmenopausal Women: Age-Stratified Multivariate Cox Regression eTable 6. Sensitivity Analysis: Multivariate Cox Regression Excluding Participants Who Were Taking Cardiovascular Drugs at Baseline eTable 7. Sensitivity Analysis: Multivariate Cox Regression Excluding Participants Who Had Cardiovascular Diseases at Baseline eTable 8. Sensitivity Analysis: Multivariate Cox Regression Excluding Participants Who Developed More Than 1 Subtype of Stroke During Follow-up eTable 9. Characteristics of Parous Postmenopausal Women at Baseline eTable 10. Sensitivity Analysis: Lactation Duration and Prevalent Stroke Among Parous Postmenopausal Women in the CKB Study: Logistic Regression at Baseline eFigure 1. Incidence Density of Stroke and its Subtypes eFigure 2. The Age-Stratified Dose-Risk Association of Lifetime Lactation Duration With Stroke and Its Subtypes in Parous Postmenopausal Women eFigure 3. The Age-Stratified Dose-Risk Association of Mean Lactation Duration Per Child With Stroke and Its Subtypes in Parous Postmenopausal Women eFigure 4. The Age-Stratified Dose-Risk Association of Lactation Duration for the First Child With Stroke and Its Subtypes in Parous Postmenopausal Women [file jamanetwopen-e220437-s001.pdf]

Supplemental Online Content

Ren Z, Yi Q, Hou L, et al. Lactation duration and the risk of subtypes of stroke among parous postmenopausal women from the China Kadoorie Biobank. *JAMA Netw Open*. 2022;5(2):e220437.
doi:10.1001/jamanetworkopen.2022.0437

eTable 1. Characteristics of Parous Postmenopausal Women Without Prior Stroke at Baseline
eTable 2. Incidence Density of Stroke And Its Subtypes Among Parous Postmenopausal Women
eTable 3. Lifetime Lactation Duration and the Risk of Stroke and Subtypes Among Parous Postmenopausal Women: Age-Stratified Multivariate Cox Regression
eTable 4. Mean Lactation Duration per Child and the Risk of Stroke and Subtypes Among Parous Postmenopausal Women: Age-Stratified Multivariate Cox Regression
eTable 5. Lactation Duration for the First Child and the Risk of Stroke and Subtypes Among Parous Postmenopausal Women: Age-Stratified Multivariate Cox Regression
eTable 6. Sensitivity Analysis: Multivariate Cox Regression Excluding Participants Who Were Taking Cardiovascular Drugs at Baseline
eTable 7. Sensitivity Analysis: Multivariate Cox Regression Excluding Participants Who Had Cardiovascular Diseases at Baseline
eTable 8. Sensitivity Analysis: Multivariate Cox Regression Excluding Participants Who Developed More Than 1 Subtype of Stroke During Follow-up
eTable 9. Characteristics of Parous Postmenopausal Women at Baseline
eTable 10. Sensitivity Analysis: Lactation Duration and Prevalent Stroke Among Parous Postmenopausal Women in the CKB Study: Logistic Regression at Baseline
eFigure 1. Incidence Density of Stroke and its Subtypes
eFigure 2. The Age-Stratified Dose-Risk Association of Lifetime Lactation Duration With Stroke and Its Subtypes in Parous Postmenopausal Women
eFigure 3. The Age-Stratified Dose-Risk Association of Mean Lactation Duration Per Child With Stroke and Its Subtypes in Parous Postmenopausal Women
eFigure 4. The Age-Stratified Dose-Risk Association of Lactation Duration for the First Child With Stroke and Its Subtypes in Parous Postmenopausal Women

This supplemental material has been provided by the authors to give readers additional information about their work.

© 2022 Ren Z et al. *JAMA Network Open.*

eTable 1. Characteristics of Parous Postmenopausal Women Without Prior Stroke at Baseline

**Baseline Characteristics**	**Non-stroke during follow-up (*N*=113,790)**	**New-onset stroke (*N*=15,721)**		
		**Ischemic stroke (*N*=13,427)**	**Intracerebral hemorrhage (*N*=2,567)**	**Subarachnoid hemorrhage (*N*=284)**
Age, year	57.8 (53.8-63.8)	62.5 (56.5-68.4)	63.1 (56.8-69.1)	60.6 (55.4-67.2)
Highest education completed				
Primary school or less	83079 (73.0)	8962 (66.8)	2191 (85.4)	208 (73.2)
Middle or high school	27783 (24.4)	3801 (28.3)	340 (13.3)	67 (23.6)
Technical school/college or above	2928 (2.6)	664 (5.0)	36 (1.4)	9 (3.2)
Occupation				
Farmer or worker	46776 (41.1)	3616 (26.9)	1072 (41.8)	101 (35.6)
Sales and others[a]	5903 (5.2)	412 (3.1)	40 (1.6)	6 (2.1)
Professionals	1598 (1.4)	158 (1.2)	7 (0.3)	4 (1.4)
Retired and others[b]	59513 (52.3)	9241 (68.8)	1448 (56.4)	173 (60.9)
Household income, yuan				
0-4999	15038 (13.2)	1817 (13.5)	601 (23.4)	34 (12.0)
5000-19999	53015 (46.6)	6811 (50.7)	1342 (52.3)	143 (50.4)
≥20000	45737 (40.2)	4799 (35.7)	624 (24.3)	107 (37.7)
Residence				
Rural	60756 (53.4)	5468 (40.7)	1855 (72.3)	153 (53.9)
Urban	53034 (46.6)	7959 (59.3)	712 (27.7)	131 (46.1)
Marital status				
Married	95871 (84.3)	10541 (78.5)	1930 (75.2)	232 (81.7)

© 2022 Ren Z et al. *JAMA Network Open.*

Baseline Characteristics		Non-stroke during follow-up (*N*=113,790)	New-onset stroke (*N*=15,721)		
			**Ischemic stroke (*N*=13,427)**	**Intracerebral hemorrhage (*N*=2,567)**	**Subarachnoid hemorrhage (*N*=284)**
	Unmarried	17919 (15.8)	2886 (21.5)	637 (24.8)	52 (18.3)
Smoking status					
	Never smoking	106242 (93.4)	12317 (91.7)	2323 (90.5)	259 (91.2)
	Ever smoker	7548 (6.6)	1110 (8.3)	244 (9.5)	25 (8.8)
Passive smoking history					
	Yes	61823 (54.3)	6626 (49.4)	1415 (55.1)	133 (46.8)
	No	51967 (45.7)	6801 (50.7)	1152 (44.9)	151 (53.2)
Drinking status					
	Never drinking	107727 (94.7)	12754 (95.0)	2424 (94.4)	264 (93.0)
	Ever drinker	6063 (5.3)	673 (5.0)	143 (5.6)	20 (7.0)
Physical activity, MET-hours/day		14.0 (8.9-22.4)	11.2 (8.4-15.5)	11.2 (8.4-19.0)	11.7 (8.4-17.7)
BMI, kg/m^2		23.7 (21.4-26.2)	24.5 (22.2-27.1)	23.5 (21.0-26.3)	24.0 (21.5-26.5)
Waist circumference, cm		80.1 (73.3-87.0)	82.7 (76.1-90.0)	80.6 (73.1-88.0)	81.0 (73.4-88.1)
Age of menopause, year		50.0 (48.0-51.0)	50.0 (48.0-52.0)	50.0 (48.0-51.0)	50.0 (48.0-52.0)
Gravidity					
	≤3	55254 (48.6)	5016 (37.4)	805 (31.4)	114 (40.1)
	4	25299 (22.2)	3078 (22.9)	549 (21.4)	62 (21.8)
	≥5	33237 (29.2)	5333 (39.7)	1213 (47.3)	108 (38.0)
Live birth counts					
	1	19585 (17.2)	1549 (11.5)	179 (7.0)	40 (14.1)
	2	37048 (32.6)	3568 (26.6)	497 (19.4)	83 (29.2)

© 2022 Ren Z et al. *JAMA Network Open.*

**Baseline Characteristics**	**Non-stroke during follow-up (*N*=113,790)**	**New-onset stroke (*N*=15,721)**		
		**Ischemic stroke (*N*=13,427)**	**Intracerebral hemorrhage (*N*=2,567)**	**Subarachnoid hemorrhage (*N*=284)**
≥3	57157 (50.2)	8310 (61.9)	1891 (73.7)	161 (56.7)
Lifetime lactation duration, months	36.0 (24.0-60.0)	42.0 (24.0-70.0)	54.0 (36.0-84.0)	36.0 (24.0-64.5)
Mean lactation duration per child, months	12.0 (12.0-18.5)	12.7 (12.0-19.5)	15.0 (12.0-22.0)	12.0 (12.0-18.0)
Lactation duration for the first child, months	12.0 (12.0-18.0)	12.0 (12.0-18.0)	13.0 (12.0-24.0)	12.0 (12.0-18.0)
Baseline history of diabetes mellitus				
Yes	5389 (4.7)	13694 (90.2)	199 (7.8)	16 (5.6)
No	108401 (95.3)	1486 (9.8)	2368 (92.3)	268 (94.4)
Baseline history of hypertension				
Yes	43806 (38.5)	7057 (52.6)	1793 (69.9)	159 (56.0)
No	69984 (61.5)	6370 (47.4)	774 (30.2)	125 (44.0)
History of cancer				
Yes	827 (0.7)	127 (1.0)	18 (0.7)	6 (2.1)
No	112963 (99.3)	13300 (99.1)	2549 (99.3)	278 (97.9)
History of taking contraceptive pills				
Yes	12166 (10.7)	1032 (7.7)	149 (5.8)	31 (10.9)
No	101624 (89.3)	12395 (92.3)	2418 (94.2)	253 (89.1)
History of anticoagulation therapy				
Yes	1346 (1.2)	300 (2.2)	72 (2.8)	3 (1.1)
No	112444 (98.8)	13127 (97.8)	2495 (97.2)	281 (98.9)
History of hypolipidemic therapy				
Yes	347 (0.3)	71 (0.5)	19 (0.7)	1 (0.4)

© 2022 Ren Z et al. *JAMA Network Open.*

Baseline Characteristics		Non-stroke during follow-up (*N*=113,790)	New-onset stroke (*N*=15,721)		
			Ischemic stroke (*N*=13,427)	Intracerebral hemorrhage (*N*=2,567)	Subarachnoid hemorrhage (*N*=284)
	No	113443 (99.7)	13356 (99.5)	2548 (99.3)	283 (99.7)

Notes: Values are presented as number (N) with percent (%) or medians with interquartile ranges (IQRs). [a] Sales and others: Including sales, self-employed and people in other un-specified occupations. [b] Retired and others: Including those retired, un-employed and home makers.

© 2022 Ren Z et al. *JAMA Network Open.*

eTable 2. Incidence Density of Stroke And Its Subtypes Among Parous Postmenopausal Women

	Total stroke	Ischemic stroke	Intracerebral hemorrhage	Subarachnoid hemorrhage
Case(n)	15721	13427	2567	284
Lifetime lactation duration				
0 month	2011.7	1836.9	188.2	26.2
< 7 months	1289.6	1168.6	105.4	33.7
7-12 months	900.8	816.8	91.2	15.0
13-18 months	1116.8	992.4	99.7	26.0
19-24 months	1029.9	913.7	117.2	25.6
> 24 months	1601.1	1334.3	283.3	25.0
Mean lactation duration per child				
0 month	2011.7	1836.9	188.2	26.2
< 7 months	1561.8	1380.4	165.8	31.5
7-12 months	1270.1	1106.2	166.6	24.6
13-18 months	1455.2	1228.3	244.1	24.5
19-24 months	1582.5	1281.7	323.4	22.7
> 24 months	1479.4	1224.1	276.2	22.5
Lactation duration for the first child				
0 month	1859.3	1622.9	239.2	27.8
< 7 months	1575.2	1372.6	205.0	27.3
7-12 months	1281.9	1107.3	180.0	23.6
13-18 months	1476.5	1250.4	240.1	26.1
19-24 months	1552.2	1263.2	315.2	24.6
> 24 months	1409.6	1161.9	261.0	19.3

Note: Incidence density was expressed in 100000 person-years

© 2022 Ren Z et al. *JAMA Network Open.*

eTable 3. Lifetime Lactation Duration and the Risk of Stroke and Subtypes Among Parous Postmenopausal Women: Age-Stratified Multivariate Cox Regression

**Age at baseline, years**	**No. of cases**	**0 month**	**< 7 months**	**7-12 months**	**13-18 months**	**19-24 months**	**> 24 months**
		**HR (95% CI)**					
Total stroke							
45-54	2843	1.00 (0.84~1.19)	0.78 (0.65~0.93)	0.55 (0.50~0.61)	0.61 (0.54~0.68)	0.42 (0.39~0.47)	0.63 (0.57~0.69)
55-59	3433	1.00 (0.80~1.25)	0.80 (0.63~1.00)	0.66 (0.58~0.76)	0.62 (0.54~0.71)	0.45 (0.42~0.49)	0.52 (0.48~0.56)
60-65	3133	1.00 (0.79~1.27)	0.82 (0.60~1.13)	0.73 (0.60~0.88)	0.60 (0.49~0.73)	0.55 (0.50~0.60)	0.47 (0.44~0.50)
66-69	3416	1.00 (0.81~1.23)	0.78 (0.56~1.09)	0.77 (0.62~0.95)	0.93 (0.76~1.13)	0.68 (0.60~0.76)	0.54 (0.50~0.58)
More than 70	2896	1.00 (0.79~1.27)	0.80 (0.52~1.25)	0.73 (0.56~0.94)	0.72 (0.55~0.94)	0.80 (0.68~0.93)	0.63 (0.58~0.68)
Ischemic stroke							
45-54	2419	1.00 (0.83~1.21)	0.78 (0.65~0.94)	0.55 (0.49~0.61)	0.60 (0.53~0.68)	0.42 (0.38~0.47)	0.63 (0.57~0.70)
55-59	2965	1.00 (0.79~1.26)	0.79 (0.62~1.00)	0.66 (0.58~0.76)	0.62 (0.54~0.72)	0.46 (0.43~0.50)	0.52 (0.48~0.56)
60-65	2680	1.00 (0.78~1.29)	0.73 (0.51~1.03)	0.72 (0.59~0.89)	0.56 (0.45~0.70)	0.54 (0.49~0.60)	0.49 (0.45~0.53)
66-69	2936	1.00 (0.81~1.24)	0.75 (0.53~1.07)	0.75 (0.60~0.94)	0.88 (0.71~1.09)	0.65 (0.57~0.73)	0.52 (0.48~0.56)
More than 70	2427	1.00 (0.78~1.28)	0.86 (0.55~1.34)	0.65 (0.49~0.87)	0.66 (0.49~0.88)	0.74 (0.62~0.88)	0.74 (0.62~0.88)
Intracerebral hemorrhage							
45-54	452	1.00 (0.57~1.77)	0.68 (0.36~1.28)	0.59 (0.43~0.80)	0.55 (0.38~0.78)	0.37 (0.28~0.47)	0.62 (0.51~0.76)
55-59	526	1.00 (0.52~1.93)	0.68 (0.31~1.53)	0.63 (0.40~0.99)	0.39 (0.23~0.65)	0.33 (0.26~0.42)	0.42 (0.36~0.50)
60-65	509	1.00 (0.48~2.10)	1.38 (0.62~3.09)	1.08 (0.65~1.80)	0.81 (0.46~1.42)	0.68 (0.51~0.90)	0.42 (0.35~0.50)
66-69	552	1.00 (0.45~2.23)	1.10 (0.36~3.42)	1.36 (0.70~2.63)	1.01 (0.45~2.25)	1.34 (0.94~1.89)	1.05 (0.89~1.25)
More than 70	528	1.00 (0.52~1.93)	NA	0.69 (0.33~1.43)	0.86 (0.40~1.81)	1.08 (0.72~1.62)	0.67 (0.56~0.80)

Notes: HR, hazard ratio; CI, confidence interval. HR was adjusted for age, gravidity, age of menopause, live birth counts, diabetes, hypertension, cancer, taking contraceptive pills, anticoagulation therapy, hypolipidemic therapy, education, income, marital status, occupation, residence, smoking, passive smoking, drinking, metabolic equivalent, body mass index, and waist circumference.

© 2022 Ren Z et al. *JAMA Network Open.*

eTable 4. Mean Lactation Duration per Child and the Risk of Stroke and Subtypes Among Parous Postmenopausal Women: Age-Stratified Multivariate Cox Regression

**Age at baseline, years**	**No. of cases**	**0 month**	**< 7 months**	**7-12 months**	**13-18 months**	**19-24 months**	**> 24 months**
		**HR (95% CI)**					
Total stroke							
45-54	2843	1.00 (0.84~1.20)	0.76 (0.65~0.88)	0.47 (0.44~0.50)	0.63 (0.58~0.68)	0.72 (0.65~0.79)	0.80 (0.71~0.89)
55-59	3433	1.00 (0.80~1.25)	0.64 (0.54~0.75)	0.48 (0.45~0.51)	0.57 (0.54~0.61)	0.67 (0.62~0.73)	0.62 (0.55~0.69)
60-65	3133	1.00 (0.79~1.27)	0.79 (0.67~0.93)	0.47 (0.44~0.50)	0.52 (0.49~0.56)	0.58 (0.53~0.63)	0.54 (0.48~0.60)
66-69	3416	1.00 (0.81~1.23)	0.85 (0.72~0.99)	0.57 (0.54~0.61)	0.60 (0.56~0.64)	0.56 (0.51~0.61)	0.55 (0.49~0.62)
More than 70	2896	1.00 (0.78~1.28)	0.71 (0.59~0.86)	0.66 (0.62~0.70)	0.66 (0.61~0.71)	0.65 (0.60~0.71)	0.63 (0.55~0.71)
Ischemic stroke							
45-54	2419	1.00 (0.83~1.21)	0.76 (0.65~0.89)	0.47 (0.43~0.50)	0.63 (0.58~0.68)	0.72 (0.65~0.80)	0.78 (0.69~0.88)
55-59	2965	1.00 (0.79~1.26)	0.66 (0.55~0.78)	0.49 (0.46~0.52)	0.58 (0.54~0.62)	0.66 (0.60~0.72)	0.65 (0.58~0.73)
60-65	2680	1.00 (0.77~1.29)	0.77 (0.65~0.91)	0.48 (0.45~0.51)	0.54 (0.50~0.58)	0.58 (0.53~0.63)	0.57 (0.50~0.64)
66-69	2936	1.00 (0.81~1.24)	0.84 (0.71~1.00)	0.55 (0.52~0.58)	0.58 (0.54~0.62)	0.53 (0.49~0.58)	0.54 (0.47~0.61)
More than 70	2427	1.00 (0.77~1.29)	0.68 (0.56~0.84)	0.64 (0.60~0.68)	0.63 (0.58~0.69)	0.62 (0.56~0.68)	0.61 (0.53~0.70)
Intracerebral hemorrhage							
45-54	452	1.00 (0.56~1.78)	0.61 (0.38~0.99)	0.43 (0.36~0.52)	0.61 (0.51~0.74)	0.72 (0.59~0.88)	0.80 (0.63~1.02)
55-59	526	1.00 (0.52~1.94)	0.40 (0.23~0.70)	0.35 (0.29~0.41)	0.45 (0.38~0.53)	0.57 (0.49~0.68)	0.40 (0.31~0.52)
60-65	509	1.00 (0.47~2.11)	0.94 (0.62~1.42)	0.46 (0.39~0.54)	0.46 (0.39~0.55)	0.60 (0.50~0.71)	0.44 (0.34~0.58)
66-69	552	1.00 (0.45~2.24)	0.95 (0.56~1.62)	1.09 (0.95~1.26)	1.15 (0.98~1.35)	1.11 (0.92~1.34)	1.01 (0.77~1.32)
More than 70	528	1.00 (0.52~1.94)	0.64 (0.39~1.04)	0.65 (0.55~0.75)	0.74 (0.62~0.87)	0.79 (0.66~0.94)	0.67 (0.51~0.89)

Notes: HR, hazard ratio; CI, confidence interval. HR was adjusted for age, gravidity, age of menopause, live birth counts, diabetes, hypertension, cancer, taking contraceptive pills, anticoagulation therapy, hypolipidemic therapy, education, income, marital status, occupation, residence, smoking, passive smoking, drinking, metabolic equivalent, body mass index, and waist circumference.

© 2022 Ren Z et al. *JAMA Network Open.*

eTable 5. Lactation Duration for the First Child and the Risk of Stroke and Subtypes Among Parous Postmenopausal Women: Age-Stratified Multivariate Cox Regression

**Age at baseline, years**	**No. of cases**	**0 month**	**< 7 months**	**7-12 months**	**13-18 months**	**19-24 months**	**> 24 months**
		**HR (95% CI)**					
Total stroke							
45-54	2843	1.00 (0.86~1.16)	0.68 (0.59~0.78)	0.49 (0.46~0.52)	0.64 (0.59~0.69)	0.73 (0.66~0.80)	0.75 (0.65~0.85)
55-59	3433	1.00 (0.86~1.17)	0.66 (0.58~0.75)	0.54 (0.51~0.57)	0.70 (0.65~0.75)	0.70 (0.64~0.76)	0.70 (0.61~0.80)
60-65	3133	1.00 (0.86~1.16)	0.67 (0.60~0.77)	0.48 (0.45~0.51)	0.56 (0.52~0.61)	0.55 (0.50~0.60)	0.53 (0.45~0.61)
66-69	3416	1.00 (0.87~1.15)	0.66 (0.59~0.75)	0.58 (0.55~0.61)	0.65 (0.60~0.70)	0.60 (0.55~0.65)	0.51 (0.45~0.59)
More than 70	2896	1.00 (0.86~1.16)	0.59 (0.51~0.69)	0.62 (0.59~0.66)	0.64 (0.59~0.70)	0.63 (0.58~0.69)	0.55 (0.47~0.64)
Ischemic stroke							
45-54	2419	1.00 (0.85~1.17)	0.68 (0.59~0.78)	0.48 (0.45~0.52)	0.65 (0.60~0.71)	0.71 (0.64~0.79)	0.74 (0.64~0.85)
55-59	2965	1.00 (0.85~1.18)	0.64 (0.55~0.73)	0.52 (0.49~0.55)	0.67 (0.62~0.73)	0.67 (0.61~0.74)	0.68 (0.58~0.79)
60-65	2680	1.00 (0.85~1.17)	0.65 (0.57~0.74)	0.45 (0.43~0.48)	0.56 (0.52~0.61)	0.53 (0.48~0.59)	0.51 (0.43~0.60)
66-69	2936	1.00 (0.86~1.16)	0.65 (0.57~0.74)	0.56 (0.53~0.60)	0.63 (0.58~0.68)	0.56 (0.51~0.62)	0.50 (0.43~0.58)
More than 70	2427	1.00 (0.85~1.18)	0.60 (0.51~0.71)	0.63 (0.59~0.67)	0.63 (0.57~0.69)	0.63 (0.57~0.70)	0.56 (0.47~0.66)
Intracerebral hemorrhage							
45-54	452	1.00 (0.66~1.52)	0.56 (0.37~0.84)	0.51 (0.44~0.61)	0.61 (0.50~0.75)	0.87 (0.71~1.06)	0.73 (0.54~0.98)
55-59	526	1.00 (0.65~1.54)	0.79 (0.56~1.13)	0.61 (0.52~0.70)	0.85 (0.72~1.01)	0.87 (0.72~1.05)	0.78 (0.56~1.08)
60-65	509	1.00 (0.67~1.49)	0.91 (0.66~1.24)	0.61 (0.53~0.71)	0.57 (0.47~0.69)	0.67 (0.55~0.82)	0.70 (0.50~0.96)
66-69	552	1.00 (0.66~1.52)	0.90 (0.64~1.28)	0.93 (0.82~1.06)	0.97 (0.80~1.17)	1.01 (0.84~1.22)	0.77 (0.56~1.07)
More than 70	528	1.00 (0.69~1.44)	0.55 (0.37~0.83)	0.62 (0.54~0.72)	0.82 (0.68~0.99)	0.74 (0.62~0.89)	0.59 (0.42~0.82)

Notes: HR, hazard ratio; CI, confidence interval. HR was adjusted for age, gravidity, age of menopause, live birth counts, diabetes, hypertension, cancer, taking contraceptive pills, anticoagulation therapy, hypolipidemic therapy, education, income, marital status, occupation, residence, smoking, passive smoking, drinking, metabolic equivalent, body mass index, and waist circumference.

© 2022 Ren Z et al. *JAMA Network Open.*

eTable 6. Sensitivity Analysis: Multivariate Cox Regression Excluding Participants Who Were Taking Cardiovascular Drugs at Baseline

Lactation duration		Total stroke			Ischemic stroke			Intracerebral hemorrhage			Subarachnoid hemorrhage	
		**Cases**	**HR (95% CI)**		**Cases**	**HR (95% CI)**		**Cases**	**HR (95% CI)**		**Cases**	**HR (95% CI)**
Lifetime lactation duration												
	0 month	373	1.00 (0.90~1.11)		339	1.00 (0.90~1.11)		47	1.00 (0.73~1.36)		4	1.00 (0.37~2.67)
	< 7 months	267	0.84 (0.74~0.95)		243	0.83 (0.73~0.94)		33	0.77 (0.51~1.17)		5	1.32 (0.54~3.20)
	7-12 months	863	0.61 (0.57~0.66)		781	0.60 (0.56~0.65)		104	0.72 (0.58~0.89)		17	0.99 (0.59~1.66)
	13-18 months	656	0.66 (0.61~0.71)		590	0.66 (0.6~0.71)		69	0.52 (0.40~0.69)		16	1.34 (0.81~2.20)
	19-24 months	1575	0.51 (0.49~0.54)		1406	0.51 (0.49~0.54)		205	0.45 (0.39~0.53)		40	1.04 (0.77~1.39)
	> 24 months	9809	0.55 (0.53~0.57)		8208	0.56 (0.53~0.58)		2133	0.52 (0.48~0.57)		163	0.77 (0.59~1.02)
Mean lactation duration per child												
	0 month	373	1.00 (0.90~1.11)		339	1.00 (0.90~1.11)		40	1.00 (0.73~1.37)		4	1.00 (0.37~2.7)
	< 7 months	651	0.76 (0.7~0.82)		579	0.77 (0.71~0.83)		72	0.63 (0.50~0.80)		12	1.24 (0.70~2.21)
	7-12 months	5363	0.52 (0.51~0.53)		4698	0.53 (0.51~0.54)		706	0.47 (0.43~0.51)		110	0.95 (0.77~1.16)
	13-18 months	3266	0.59 (0.57~0.61)		2761	0.59 (0.57~0.62)		565	0.55 (0.51~0.60)		59	0.91 (0.71~1.17)
	19-24 months	2563	0.62 (0.60~0.65)		2090	0.62 (0.59~0.65)		534	0.63 (0.58~0.69)		40	0.85 (0.62~1.16)
	> 24 months	1327	0.61 (0.58~0.65)		1100	0.62 (0.59~0.66)		254	0.55 (0.49~0.63)		20	0.76 (0.49~1.19)
Lactation duration for the first child												
	0 month	767	1.00 (0.93~1.07)		666	1.00 (0.93~1.08)		111	1.00 (0.83~1.21)		11	1.00 (0.55~1.81)
	< 7 months	938	0.66 (0.62~0.70)		826	0.66 (0.62~0.71)		120	0.65 (0.54~0.78)		16	0.81 (0.50~1.33)
	7-12 months	5903	0.54 (0.52~0.55)		5129	0.53 (0.52~0.55)		833	0.59 (0.55~0.63)		115	0.72 (0.59~0.87)
	13-18 months	2808	0.65 (0.62~0.67)		2378	0.64 (0.62~0.67)		478	0.71 (0.65~0.78)		53	0.80 (0.61~1.04)
	19-24 months	2254	0.63 (0.60~0.65)		1845	0.62 (0.59~0.65)		465	0.73 (0.67~0.81)		39	0.74 (0.54~1.02)
	> 24 months	873	0.60 (0.56~0.64)		723	0.60 (0.56~0.64)		164	0.65 (0.55~0.76)		11	0.49 (0.27~0.90)

© 2022 Ren Z et al. *JAMA Network Open.*

Notes: HR, hazard ratio; CI, confidence interval. HR was adjusted for age, gravidity, age of menopause, live birth counts, diabetes, hypertension, cancer, taking contraceptive pills, education, income, marital status, occupation, residence, smoking, passive smoking, drinking, metabolic equivalent, body mass index, and waist circumference.

© 2022 Ren Z et al. *JAMA Network Open.*

eTable 7. Sensitivity Analysis: Multivariate Cox Regression Excluding Participants Who Had Cardiovascular Diseases at Baseline

Lactation duration	Total stroke		Ischemic stroke		Intracerebral hemorrhage		Subarachnoid hemorrhage	
	**Cases**	**HR (95% CI)**	**Cases**	**HR (95% CI)**	**Cases**	**HR (95% CI)**	**Cases**	**HR (95% CI)**
Lifetime lactation duration								
0 month	330	1.00 (0.90~1.11)	301	1.00 (0.89~1.12)	38	1.00 (0.73~1.38)	5	1.00 (0.42~2.41)
< 7 months	240	0.79 (0.70~0.90)	216	0.76 (0.67~0.87)	22	0.77 (0.51~1.17)	7	1.43 (0.67~3.03)
7-12 months	849	0.62 (0.58~0.67)	765	0.60 (0.56~0.65)	95	0.73 (0.59~0.90)	17	0.74 (0.45~1.24)
13-18 months	617	0.66 (0.61~0.71)	548	0.64 (0.59~0.70)	56	0.54 (0.41~0.70)	17	1.09 (0.67~1.76)
19-24 months	1632	0.53 (0.51~0.56)	1437	0.53 (0.50~0.55)	205	0.53 (0.46~0.60)	43	0.79 (0.59~1.05)
> 24 months	10027	0.56 (0.54~0.58)	8334	0.56 (0.54~0.59)	1931	0.55 (0.50~0.60)	168	0.54 (0.41~0.71)
Mean lactation duration per child								
0 month	330	1.00 (0.90~1.12)	301	1.00 (0.89~1.12)	38	1.00 (0.73~1.38)	5	1.00 (0.41~2.43)
< 7 months	598	0.74 (0.68~0.80)	524	0.73 (0.67~0.80)	72	0.64 (0.51~0.81)	15	1.18 (0.70~1.97)
7-12 months	5532	0.54 (0.52~0.55)	4810	0.53 (0.52~0.55)	781	0.50 (0.47~0.54)	120	0.71 (0.59~0.87)
13-18 months	3248	0.59 (0.57~0.61)	2722	0.59 (0.57~0.61)	602	0.58 (0.54~0.63)	59	0.64 (0.50~0.82)
19-24 months	2629	0.64 (0.61~0.66)	2124	0.62 (0.60~0.65)	580	0.67 (0.62~0.73)	38	0.56 (0.41~0.78)
> 24 months	1358	0.63 (0.59~0.66)	1120	0.63 (0.59~0.67)	274	0.58 (0.51~0.65)	20	0.53 (0.34~0.83)
Lactation duration for the first child								
0 month	722	1.00 (0.93~1.08)	626	1.00 (0.92~1.08)	108	1.00 (0.83~1.21)	12	1.00 (0.57~1.77)
< 7 months	898	0.66 (0.61~0.70)	781	0.65 (0.60~0.69)	125	0.68 (0.57~0.81)	19	0.87 (0.56~1.37)
7-12 months	6090	0.56 (0.54~0.57)	5251	0.55 (0.53~0.56)	921	0.64 (0.59~0.68)	124	0.67 (0.56~0.81)
13-18 months	2776	0.65 (0.63~0.68)	2329	0.64 (0.62~0.67)	503	0.74 (0.68~0.80)	54	0.72 (0.55~0.94)
19-24 months	2332	0.65 (0.63~0.68)	1894	0.63 (0.61~0.66)	516	0.81 (0.74~0.88)	35	0.58 (0.42~0.82)
> 24 months	877	0.61 (0.57~0.65)	720	0.60 (0.56~0.65)	174	0.68 (0.58~0.79)	13	0.52 (0.30~0.90)

© 2022 Ren Z et al. *JAMA Network Open.*

Notes: HR, hazard ratio; CI, confidence interval. HR was adjusted for age, gravidity, age of menopause, live birth counts, diabetes, hypertension, cancer, taking contraceptive pills, anticoagulation therapy, hypolipidemic therapy, education, income, marital status, occupation, residence, smoking, passive smoking, drinking, metabolic equivalent, body mass index, and waist circumference.

© 2022 Ren Z et al. *JAMA Network Open.*

eTable 8. Sensitivity Analysis: Multivariate Cox Regression Excluding Participants Who Developed More Than 1 Subtype of Stroke During Follow-up

Lactation duration		Total stroke			Ischemic stroke			Intracerebral hemorrhage			Subarachnoid hemorrhage	
		Cases	HR (95% CI)		Cases	HR (95% CI)		Cases	HR (95% CI)		Cases	HR (95% CI)
Lifetime lactation duration												
	0 month	394	1.00 (0.91~1.10)		359	1.00 (0.90~1.11)		27	1.00 (0.69~1.46)		3	1.00 (0.32~3.11)
	< 7 months	265	0.80 (0.71~0.90)		241	0.79 (0.69~0.89)		17	0.92 (0.57~1.48)		3	1.04 (0.33~3.24)
	7-12 months	921	0.63 (0.59~0.67)		833	0.61 (0.57~0.66)		67	0.83 (0.65~1.06)		14	1.00 (0.56~1.78)
	13-18 months	700	0.67 (0.63~0.73)		626	0.66 (0.61~0.71)		52	0.77 (0.59~1.01)		10	1.07 (0.57~2.01)
	19-24 months	1708	0.53 (0.50~0.55)		1513	0.52 (0.50~0.55)		142	0.57 (0.48~0.67)		30	1.00 (0.72~1.40)
	> 24 months	10445	0.56 (0.54~0.58)		8670	0.57 (0.55~0.59)		1480	0.63 (0.57~0.69)		97	0.79 (0.49~1.10)
Mean lactation duration per child												
	0 month	394	1.00 (0.90~1.11)		359	1.00 (0.90~1.11)		27	1.00 (0.68~1.46)		3	1.00 (0.32~3.15)
	< 7 months	693	0.76 (0.70~0.82)		615	0.76 (0.70~0.82)		60	0.76 (0.59~0.98)		8	1.13 (0.56~2.28)
	7-12 months	5771	0.53 (0.52~0.55)		5019	0.53 (0.52~0.55)		577	0.57 (0.53~0.63)		78	0.93 (0.73~1.18)
	13-18 months	3495	0.60 (0.58~0.62)		2945	0.61 (0.58~0.63)		466	0.66 (0.61~0.73)		33	0.74 (0.53~1.04)
	19-24 months	2694	0.64 (0.62~0.67)		2167	0.63 (0.60~0.66)		453	0.79 (0.72~0.87)		22	0.71 (0.46~1.09)
	> 24 months	1386	0.64 (0.60~0.67)		1137	0.65 (0.61~0.68)		202	0.64 (0.56~0.74)		13	0.76 (0.43~1.32)
Lactation duration for the first child												
	0 month	813	1.00 (0.93~1.07)		710	1.00 (0.93~1.08)		82	1.00 (0.80~1.24)		7	1.00 (0.47~2.11)
	< 7 months	999	0.65 (0.61~0.69)		868	0.64 (0.59~0.68)		104	0.76 (0.63~0.93)		10	0.77 (0.41~1.45)
	7-12 months	6364	0.54 (0.53~0.55)		5489	0.53 (0.51~0.54)		692	0.67 (0.62~0.72)		78	0.73 (0.52~1.01)
	13-18 months	2968	0.64 (0.62~0.67)		2512	0.64 (0.61~0.66)		378	0.75 (0.68~0.83)		31	0.73 (0.51~1.03)
	19-24 months	2376	0.64 (0.61~0.67)		1917	0.62 (0.59~0.65)		393	0.84 (0.76~0.93)		22	0.69 (0.45~1.05)

© 2022 Ren Z et al. *JAMA Network Open.*

	> 24 months	913	0.61 (0.57~0.65)		746	0.60 (0.56~0.65)		136	0.73 (0.61~0.86)		9	0.66 (0.34~1.28)

Notes: HR, hazard ratio; CI, confidence interval. HR was adjusted for age, gravidity, age of menopause, live birth counts, diabetes, hypertension, cancer, taking contraceptive pills, anticoagulation therapy, hypolipidemic therapy, education, income, marital status, occupation, residence, smoking, passive smoking, drinking, metabolic equivalent, body mass index, and waist circumference.

© 2022 Ren Z et al. *JAMA Network Open.*

eTable 9. Characteristics of Parous Postmenopausal Women at Baseline

Baseline Characteristics		Prevalent stroke (N=2,996)	Non-stroke at baseline (*N*=129,511)
Age, year		63.4 (57.7-68.5)	58.3 (54.0-64.6)
Highest education completed			
	Primary school or less	1872 (62.5)	93984 (72.6)
	Middle or high school	984 (32.8)	31902 (24.6)
	Technical school/college or above	140 (4.7)	3625 (2.8)
Occupation			
	Farmer or worker	440 (14.7)	51352 (39.7)
	Sales and others [a]	68 (2.3)	6359 (4.9)
	Professionals	23 (0.8)	1763 (1.4)
	Retired and others [b]	2465 (82.3)	70037 (54.1)
Household income, yuan			
	0-4999	384 (12.8)	17391 (13.4)
	5000-19999	1522 (50.8)	60995 (47.1)
	≥20000	1090 (36.4)	51125 (39.5)
Residence			
	Rural	1096 (36.6)	67880 (52.4)
	Urban	1900 (63.4)	61631 (47.6)
Marital status			
	Married	2338 (78.0)	108138 (83.5)
	Unmarried	658 (22.0)	21373 (16.5)
Smoking status			
	Never smoking	2688 (89.7)	120626 (93.1)
	Ever smoker	308 (10.3)	8885 (6.9)
Passive smoking history			
	Yes	1465 (48.9)	69694 (53.8)
	No	1531 (51.1)	59817 (46.2)
Drinking status			
	Never drinking	2869 (95.8)	122651 (94.7)
	Ever drinker	127 (4.2)	6860 (5.3)
Physical activity, MET-hours/day		9.3 (6.1-12.7)	13.5 (8.9-21.7)
BMI, kg/m^2		24.9 (22.6-27.4)	23.8 (21.5-26.3）
Waist circumference, cm		84.2 (77.8-91.0)	80.4 (73.6-87.2)
Age of menopause, year		50.0 (48.0-52.0)	50.0 (48.0-51.0)
Gravidity			
	≤3	1081 (36.1)	61008 (47.1)
	4	665 (22.2)	28877 (22.3)
	≥5	1250 (41.7)	39626 (30.6)
Live birth counts			

© 2022 Ren Z et al. *JAMA Network Open.*

Baseline Characteristics		Prevalent stroke (N=2,996)	Non-stroke at baseline (*N*=129,511)
	1	287 (9.6)	21309 (16.5)
	2	836 (27.9)	41088 (31.7)
	≥3	1873 (62.5)	67114 (51.8)
Lifetime lactation duration, months		42.0 (24.0-72.0)	36.0 (24.0-60.0)
Mean lactation duration per child, months		13.1 (12.0-19.6)	12.0 (12.0-18.8)
Lactation duration for the first child, months		12.0 (11.0-18.0)	12.0 (12.0-18.0)
History of diabetes mellitus			
	Yes	494 (16.5)	6887 (5.3)
	No	2502 (83.5)	122624 (94.7)
History of hypertension			
	Yes	1757 (58.6)	52367 (40.4)
	No	1239 (41.4)	77144 (59.6)
History of cancer			
	Yes	24 (0.8)	973 (0.8)
	No	2972 (99.2)	128538 (99.3)
History of taking contraceptive pills			
	Yes	331 (11.1)	13353 (10.3)
	No	2665 (89.0)	116158 (89.7)
History of anticoagulation therapy			
	Yes	319 (10.7)	1700 (1.3)
	No	2677 (89.4)	127811 (98.7)
History of hypolipidemic therapy			
	Yes	48 (1.6)	432 (0.3)
	No	2948 (98.4)	129079 (99.7)

Notes: Values are presented as number (N) with percent (%) or medians with interquartile ranges (IQRs).
[a] Sales and others: Including sales, self-employed and people in other un-specified occupations. [b] Retired and others: Including those retired, un-employed and home makers.

© 2022 Ren Z et al. *JAMA Network Open*.

eTable 10. Sensitivity Analysis: Lactation Duration and Prevalent Stroke Among Parous Postmenopausal Women in the CKB Study: Logistic Regression at Baseline

	**0 month**	**< 7 months**	**7-12 months**	**13-18 months**	**19-24 months**	**> 24 months**
	**OR (95% CI)**					
Lifetime lactation duration						
Participants, No. (%)	2645 (2.0)	2715 (2.1)	12814 (9.7)	7781 (5.9)	21087 (15.9)	85465 (64.5)
No. of cases	92	63	166	133	393	2149
Model 1	1.00 (0.81~1.23)	0.81 (0.63~1.05)	0.49 (0.42~0.58)	0.61 (0.51~0.72)	0.59 (0.53~0.65)	0.58 (0.55~0.61)
Model 2	1.00 (0.81~1.24)	0.82 (0.64~1.06)	0.51 (0.43~0.60)	0.66 (0.56~0.79)	0.75 (0.68~0.82)	0.93 (0.86~1.01)
Model 3	1.00 (0.81~1.24)	0.82 (0.64~1.06)	0.52 (0.45~0.61)	0.69 (0.58~0.82)	0.79 (0.71~0.87)	0.97 (0.90~1.05)
Model 4	1.00 (0.81~1.24)	0.83 (0.64~1.07)	0.53 (0.45~0.62)	0.68 (0.57~0.81)	0.78 (0.71~0.86)	0.95 (0.88~1.03)
Mean lactation duration per child						
Participants, No. (%)	2645 (2.0)	5845 (4.4)	58744 (44.3)	31190 (23.5)	22080 (16.7)	12003 (9.1)
No. of cases	92	171	1142	759	523	309
Model 1	1.00 (0.81~1.23)	0.82 (0.70~0.95)	0.50 (0.48~0.54)	0.63 (0.59~0.68)	0.59 (0.54~0.65)	0.68 (0.61~0.76)
Model 2	1.00 (0.81~1.24)	0.90 (0.77~1.06)	0.64 (0.60~0.68)	0.90 (0.84~0.97)	0.98 (0.89~1.07)	1.19 (0.88~1.28)
Model 3	1.00 (0.80~1.24)	0.93 (0.79~1.09)	0.67 (0.63~0.71)	0.94 (0.87~1.01)	1.00 (0.92~1.10)	1.20 (0.96~1.27)
Model 4	1.00 (0.80~1.24)	0.93 (0.79~1.09)	0.66 (0.62~0.71)	0.92 (0.85~0.99)	0.98 (0.90~1.08)	1.17 (0.92~1.28)
Lactation duration for the first child						
Participants, No. (%)	5824 (4.4)	8425 (6.4)	64168 (48.4)	26043 (19.7)	19847 (15.0)	8200 (6.2)
No. of cases	198	237	1289	621	453	198
Model 1	1.00 (0.87~1.15)	0.86 (0.75~0.98)	0.60 (0.57~0.64)	0.75 (0.69~0.81)	0.65 (0.60~0.72)	0.73 (0.63~0.84)
Model 2	1.00 (0.86~1.16)	0.81 (0.71~0.92)	0.62 (0.59~0.66)	0.85 (0.78~0.92)	0.87 (0.79~0.96)	1.00 (0.86~1.15)
Model 3	1.00 (0.86~1.16)	0.81 (0.71~0.93)	0.63 (0.60~0.67)	0.86 (0.79~0.93)	0.87 (0.79~0.96)	0.98 (0.84~1.13)

© 2022 Ren Z et al. *JAMA Network Open.*

Model 4	1.00 (0.86~1.16)	0.81 (0.71~0.93)	0.63 (0.60~0.67)	0.85 (0.78~0.92)	0.86 (0.78~0.95)	0.97 (0.84~1.12)

Notes: OR, odds ratio; CI, confidence interval. Model 1: Adjusted for age; Model 2: Further adjusted for gravidity, age of menopause, live birth counts, diabetes, hypertension, cancer, taking contraceptive pills, anticoagulation therapy, hypolipidemic therapy, education, income, marital status, occupation, and residence based on model 1; Model 3: Further adjusted for smoking, passive smoking, drinking, and metabolic equivalent based on model 2; Model 4: Further adjusted for body mass index, and waist circumference based on model 3.

© 2022 Ren Z et al. *JAMA Network Open.*

eFigure 1. Incidence Density of Stroke and its Subtypes

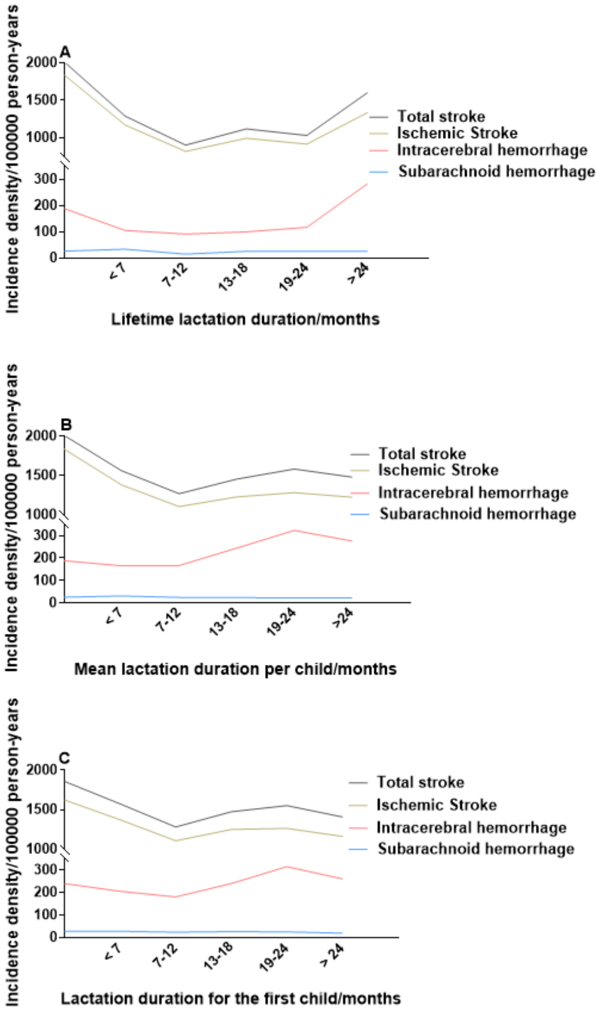

eFigure 1. Incidence density of stroke and its subtypes

A: lifetime lactation duration; B: mean lactation duration per child; C: lactation duration for the first child

© 2022 Ren Z et al. *JAMA Network Open.*

eFigure 2. The Age-Stratified Dose-Risk Association of Lifetime Lactation Duration With Stroke and Its Subtypes in Parous Postmenopausal Women

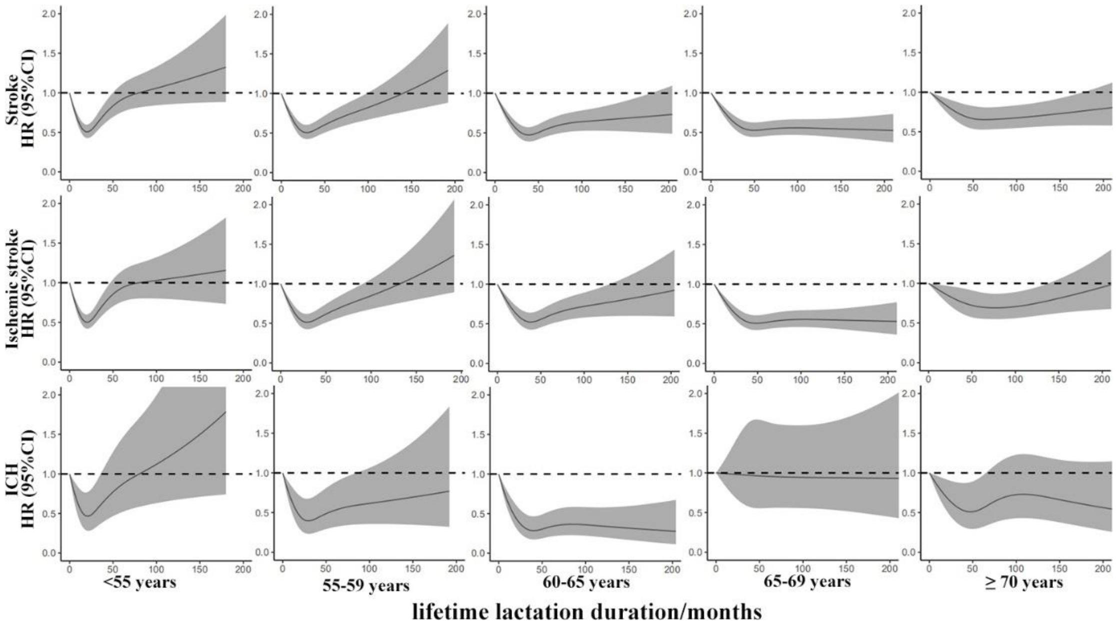

Notes: HR, hazard ratio; CI, confidence interval. HR was adjusted for age, gravidity, age of menopause, live birth counts, diabetes, hypertension, cancer, taking contraceptive pills, anticoagulation therapy, hypolipidemic therapy, education, income, marital status, occupation, residence, smoking, passive smoking, drinking, metabolic equivalent, body mass index, and waist circumference.

© 2022 Ren Z et al. *JAMA Network Open.*

eFigure 3. The Age-Stratified Dose-Risk Association of Mean Lactation Duration Per Child With Stroke and Its Subtypes in Parous Postmenopausal Women

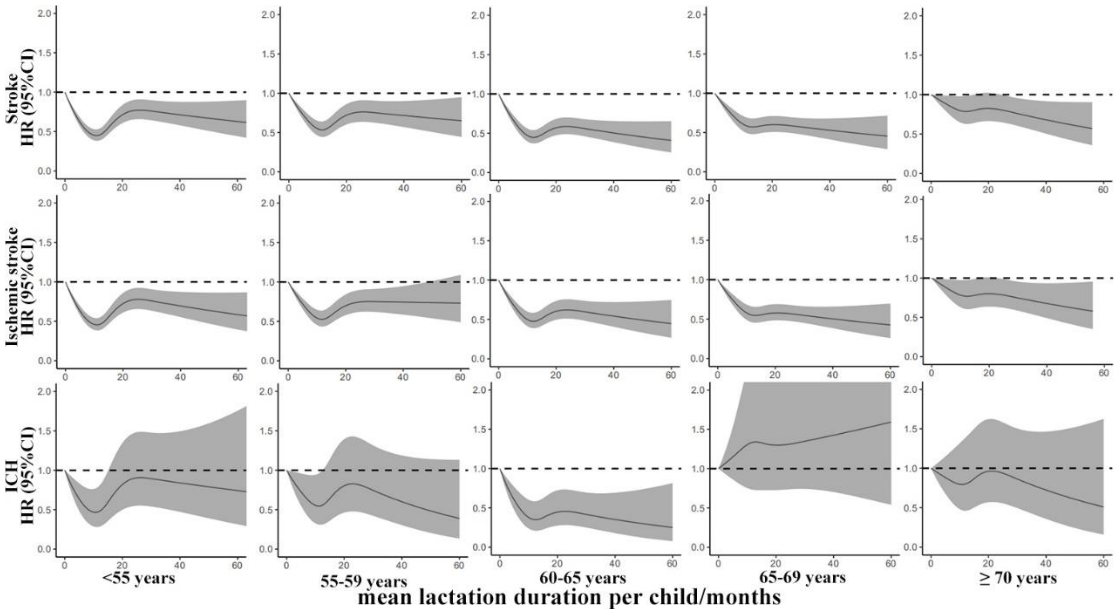

Notes: HR, hazard ratio; CI, confidence interval. HR was adjusted for age, gravidity, age of menopause, live birth counts, diabetes, hypertension, cancer, taking contraceptive pills, anticoagulation therapy, hypolipidemic therapy, education, income, marital status, occupation, residence, smoking, passive smoking, drinking, metabolic equivalent, body mass index, and waist circumference.

© 2022 Ren Z et al. *JAMA Network Open.*

eFigure 4. The Age-Stratified Dose-Risk Association of Lactation Duration for the First Child With Stroke and Its Subtypes in Parous Postmenopausal Women

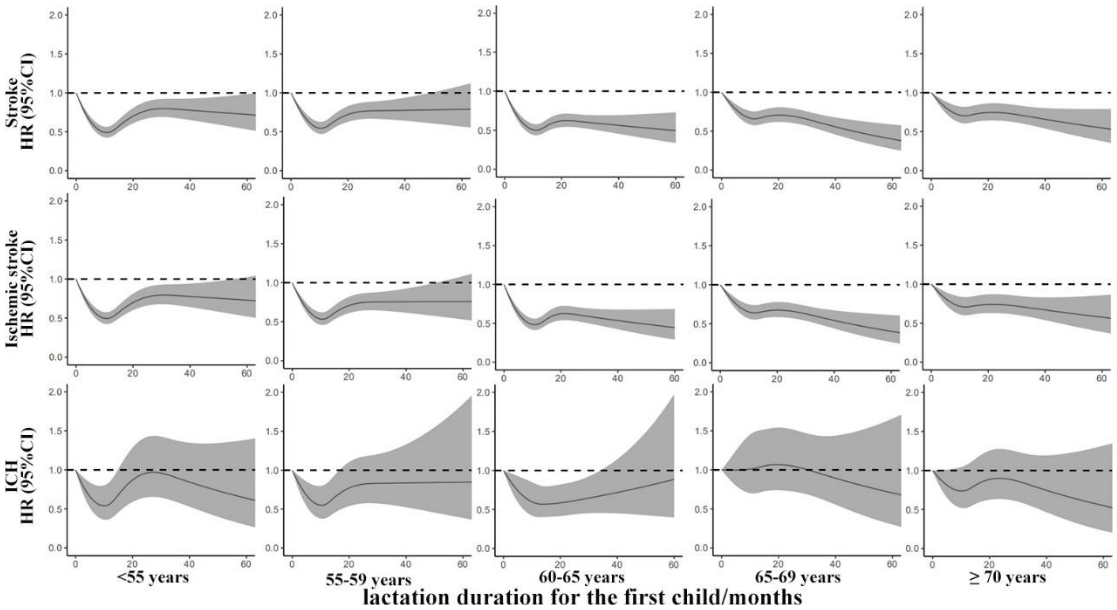

Notes: HR, hazard ratio; CI, confidence interval. HR was adjusted for age, gravidity, age of menopause, live birth counts, diabetes, hypertension, cancer, taking contraceptive pills, anticoagulation therapy, hypolipidemic therapy, education, income, marital status, occupation, residence, smoking, passive smoking, drinking, metabolic equivalent, body mass index, and waist circumference.

© 2022 Ren Z et al. *JAMA Network Open.*
